# Identifying the Risk Factors for Malignant Mammary Tumors in Dogs: A Retrospective Study

**DOI:** 10.3390/vetsci10100607

**Published:** 2023-10-05

**Authors:** Elis Maressa Gonçalves da Silva, Thaisa Reis dos Santos, Marcelo José Barbosa Silva

**Affiliations:** 1 Laboratory of Tumor Biomarkers and Osteoimmunology, Institute of Biomedical Sciences, Federal University of Uberlândia, Avenida Pará, 1720, Campus Umuarama, Uberlândia 38400-000, MG, Brazil; 2 Instituto de Ciências Clínicas Veterinárias, Universidad Austral del Chile, Valdivia 5090000, Chile

**Keywords:** mammary tumors, dogs, veterinary oncology, statistical analyses, overweight, risk factors

## Abstract

**Simple Summary:**

The increasing incidence of cancer in animals is a growing concern, with mammary cancer being the most prevalent form in female dogs. Efforts have been made to prevent and understand the factors contributing to its development. Using data from a veterinary hospital of reference in Brazil, we determined that the size dog, breed type, housing, and body score influence the risk of developing malignant mammary tumors.

**Abstract:**

Mammary cancer is one of the main causes of death in female dogs worldwide, considering that many risk factors are involved in its development. This study aimed to elucidate the relationship between epidemiological and clinical risk factors with the histopathological diagnosis of malignant mammary tumors in dogs treated at the Veterinary Hospital of the Federal University of Uberlândia, which has one of the first veterinary oncology services in Brazil. A retrospective matched case-control study was conducted to identify risk factors for the development of malignant mammary tumors in dogs. The variables analyzed were size dog, breed, housing, type of diet, and body score. Potential risk factors were selected by univariate analysis (*p* < 0.25) before multivariate forward binary logistic regression. The most frequent benign tumor was the benign mixed tumor (35.2%), and the most frequent malignant tumor was the mixed carcinoma (27.4%). Size dog, breed, housing, and overweight are predictors of malignant mammary tumors in dogs. The highest risk of developing malignant mammary tumors is associated with large female dogs, Yorkshire or Poodle breeds, living outside the home, or being overweight.

## 1. Introduction

Mammary neoplasms, the most common in female dogs, represent 50 to 70% of diagnoses [1,2,3,4,5,6]. With an annual incidence of approximately 200 cases per 100,000 dogs and a malignancy rate of over 50%, canine mammary cancer, as well as human, represents an important health problem [2,4,7,8,9]. In Brazil, the prevalence of these tumors ranges from 26 to 63%, and the malignancy index from 62% to 92% [10,11,12,13,14,15,16,17,18]. This malignancy is greater than reported worldwide. These data emphasize the importance of epidemiological studies in expanding our understanding of pathogenesis and identifying prevention and treatment strategies that benefit both species [19,20,21]. 

The etiology of canine mammary cancer is complex and multifactorial, involving epidemiological, histological, and clinical factors. Age and reproductive status are the known and most important epidemiological factors for canine mammary cancer. However, other factors, such as breed, type of diet, and obesity, have been associated with an increased risk of developing canine mammary cancer [6,22]. Despite the variation in life expectancy of different breeds, the highest occurrence of mammary tumors (MTs) is between 7 and 11 years old, with the average age of dogs with malignant tumors being higher than that of dogs with benign tumors. In general, the risk increases with age, becoming significant after eight years of age [7,23,24,25].

Exposure to endogenous sex hormones is known to play an important role in the development of the disease. Female dogs spayed before the first estrous cycle have a 0.5% lifetime relative risk of developing MTs. This value varies between 8% and 26% if they are ovariohysterectomized after the first and second heat, respectively. The protective effect of ovariohysterectomy (OHE) decreases with each estrous cycle, with the effective age around 2.5 years of age [23]. Mammary tumors affect dogs with or without a defined breed but are more commonly reported in purebred and smaller dogs [7,25,26]. 

In humans, obesity is a known risk factor for breast cancer; obese women are more often diagnosed with metastases and more aggressive tumors [27,28]. In dogs, body weight during puberty is known to have a significant effect on the risk of mammary tumors [29]. Being overweight and obese can influence the development and progression of canine mammary tumors, being associated with a higher tumor histological grade [30].

In most cases, surgical removal is the standard treatment and should always be followed by a histopathological examination [31,32]. The histopathological report must contain information such as tumor subtype, tumor grade, lymphatic invasion, surgical margins, and presence/absence of metastasis in regional lymph nodes [31,33,34]. The clinical characteristics of MTs are important in establishing a prognosis, and therefore evaluation of tumor size (T), lymph node involvement (N), and distant metastasis (M) are requirements of the malignant tumor classification system (TNM) for the staging and prognosis, created in 1980 by the World Health Organization (WHO) [35].

In this study, a retrospective statistical analysis was performed to analyze the characteristics of 861 mammary tumors diagnosed in females treated at the Veterinary Hospital of the Federal University of Uberlândia (HV-UFU). In May 2018, the hospital installed the Veterinary Oncology Clinic and Surgery Service (SECCON), which is the first veterinary oncology service in the state of Minas Gerais, and a pioneer among universities in Brazil to comply with the biosafety standards for the handling of chemotherapy required by the National Health Surveillance Agency (ANVISA) [36]. The relationship between risk factors and histopathological diagnosis was analyzed in order to assess their ability to generate data for predicting the diagnosis in dogs. The objective of this work was to investigate the possible risk factors for the development of malignant mammary tumors in dogs.

## 2. Materials and Methods

### 2.1. Ethics Aspects

This research was approved by the Ethics Committee on the Use of Animals of the Federal University of Uberlândia under reference number A009/21. The evaluation was waived due to its retrospective nature. 

### 2.2. Data Collection

This retrospective study was based on the review of 26,161 medical records of dogs treated at the Uberlândia University Veterinary Hospital between January 2015 and December 2020.

The samples were processed using the routine technique for histopathology: paraffin embedding and hematoxylin-eosin staining. All histological diagnoses were classified according to the recent publication Surgical Pathology of Tumors of Domestic Animals, Volume 2: Mammary Tumors [31]. Based on the applied classification [31], mammary tumors were classified as benign or malignant and staged accordingly to the modified TNM classification [6,25,32,35].

For staging, the following information was used: tumor size, lymph node status, and presence of metastases. The size was considered the largest diameter of the malignant tumor, and regional lymph nodes were evaluated by histopathology or fine-needle aspirate for cytology; immunohistochemistry was not used in any of the diagnoses. Three-way thoracic radiographs and abdominal ultrasonography were used to assess distant metastases.

**Figure 1:**
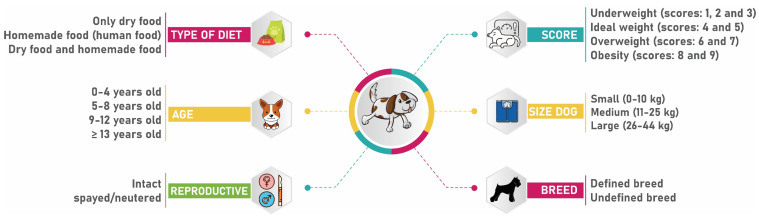
Potential clinical risk factors and their classification. The data was extracted from the medical records of dogs with 861 mammary tumors treated at the HV-UFU between January 2015 and December 2020.

Epidemiological information (Figure 1), as well as clinical characteristics of the tumor (primary size, location, metastasis, surgery, and lymph node involvement), were obtained from the records of animals with a histopathological diagnosis of mammary tumor. Animals with insufficient information in their medical records on some of the factors were excluded from the study. 

Two databases were created: the first included the patient only once in the system, with the option of subsequent diagnoses. The second included histologically different tumors from the same dog, resulting in a higher number of tumors than dogs. For statistical analysis, both databases were used, depending on the trait analyzed.

### 2.3. Statistical Analysis: Descriptive Statistics and Univariate Analysis

Statistical analysis was performed to determine risk factors associated with the development of malignant mammary tumors in dogs using the Statistical Package for the Social Sciences (SPSS) software version 26.0 (SPSS Inc., Chicago, IL, USA). Student’s *t*-test was used to analyze continuous variables, and Welch’s test correction was used when variances between groups were unequal. The Shapiro–Wilk test was used to determine normality. The chi-square (χ²) or Fisher test was used for categorical variables. The Kruskal–Wallis test, followed by Dunn’s post hoc test, was used to compare tumor size between tumor staging groups. 

The results were considered significant at *p* ≤ 0.05, and the statistical effect power was analyzed, considering a very small effect when d = 0.1; small if d = 0.2, medium if d = 0.5, large if d = 0.8, and very large if d = 2.0 [37,38]. The power of effect of the Kruskal–Wallis test was classified as follows: epsilon-squared (↋2) between 0.01 ≤ 0.08, small effect when ↋2 between 0.08 ≤ 0.26 medium effect, and when ↋2 ≥ 0.26, the effect was considered large [39,40]. 

A preliminary univariate analysis of all potential risk factors (size of dog, breed, housing, type of diet, and body score) was performed with the histopathological diagnosis of a malignant mammary tumor (Figure 1). For statistical purposes, the age variable was classified into classes (0–4 years; 5–8 years; 9–12 years, and >13 years), and the body score variable (1 to 9) was classified into four categories: underweight (scores 1, 2, and 3/9), ideal weight (scores 4 and 5/9), overweight (scores 6 and 7), and obesity (scores 8 and 9/9) [41]. 

Dogs were classified by size according to breed; mixed breed animals were classified as follows: dogs weighing less than 10 kg, small size; dogs weighing between 10 and 25 kg, medium size; dogs weighing between 26 and 44 kg, large size [42].

The results were initially described using the mean and standard deviation for continuous variables and a frequency table for categorical variables. The largest diameter of the primary tumor was treated as a continuous variable and categorized according to the tumor classification system (TNM) into three categories: T1 (<3 cm), T2 (3–5 cm), and T3 (>5 cm) [35].

### 2.4. Statistical Analysis: Multivariate Analysis and Prediction Model

A matched case-control study was conducted using logistic regression analysis to assess the impact of different variables (size of dog, breed, type of diet, housing, and body score) on the diagnosis of a malignant mammary tumor. The database used was per animal; that is, females with multiple tumors were registered only once. As previously described, age and weight were converted to categorical. For this analysis, we selected 449 female dogs with at least one diagnosis of malignant mammary tumor, confirmed by histopathological examination and with complete medical records, including histopathological reports. Controls were selected from the same population, matched by age and year of care; the following inclusion criteria were used for the case group:

- Female dogs;
- Absence of nodules or suspicion of neoplasms;
- Have not been spayed before 3 years of age.

In the process of age matching, it was found that 35 animals from the case group did not have the age information in the medical records. To address this, controls between 9 and 12 years of age were selected for matching purposes. As for the selection of controls based on reproductive status, unspayed female dogs were chosen. This decision was made because of the difficulty in finding advanced-age unspayed controls. It should be noted that female dogs spayed over 3 years of age were considered unspayed for the study, considering that the maximum recommended age for spaying to obtain preventive benefits against mammary tumors is 2.5 years [23]. 

To assess the percentage of prediction of each potential risk factor in the development of malignant mammary tumor occurrence, univariate analysis variables with a result of *p* < 0.25 were entered into a direct binary logistic regression model [43]. In the construction of the prediction model, the Hosmer-Lemeshow test was used to verify the goodness of fit of the model, and ORs greater than 1.00 with *p* < 0.05 were considered significant [44].

## 3. Results

### 3.1. Histopathological Diagnosis

Over six years, a retrospective study was conducted to determine the prevalence of mammary tumors in a population of dogs. The study population consisted of 26,161 canines whose medical records were analyzed. The incidence of mammary tumors in the population of dogs treated at the Uberlândia University Veterinary Hospital between January 2015 and December 2020 was 142 cases per 10,000 dogs (IC95%: 136 to 148). Of the 26,161 medical records analyzed, 1070 dogs with 1718 histopathological results had different tumors. Of these, 861 histologically distinct mammary tumors were detected in 465 dogs. Among these female dogs, 44% (205/465) had only one diagnosis of mammary tumor, while 55.9% (260/465) had two or more histologically different types of mammary tumor, and 88% (410/465) of females with mammary tumors have had a partial or total mastectomy. Furthermore, almost all females with mammary tumors (96.5%, 449/465) were diagnosed with at least one malignant mammary tumor.

Based on histological classification, 12.2% (105/861) of the tumors were identified as benign, while the remaining 87.8% (756/861) were classified as malignant. Furthermore, according to the applied classification scheme, 4% (36/861) of the tumors were characterized as simple benign tumors, 7.5% (65/861) as non-simple benign tumors, 0.46% (4/861) as ductal-associated benign tumors, 36.4% (314/861) as simple malignant, 41.5% (357/861) as non-simple malignant, 3% (26/861) as special type malignant and 3.1% (27/861) as sarcomas (Table 1). Of all benign tumors, the benign mixed tumor was the most commonly diagnosed lesion (37/105; 35.2%), followed by a simple adenoma (36/105; 34.2%) and a complex adenoma (24/105; 22.8%). Conversely, mixed carcinomas were the most frequently observed malignant tumors (207/756; 27.4%), followed by complex carcinomas (144/756; 19%) and tubulopapillary carcinomas (135/756; 17.8%).

### 3.2. Size Tumor, Classification of Malignant Tumors (TNM), and Staging

The following reports present the characteristics of primary mammary tumors in female dogs and their classification according to the TNM staging system. The mean size of the primary tumors was 3.63 cm (SD = 3.99, *n* = 684), with a range of 0.2–25 cm. Malignant tumors were larger (*n* = 614, mean = 3.74, SD = 4.07) than benign tumors (*n* = 70, mean = 2.62, SD = 3.05) (Diff. = 1.12 *t*-test = −2.80, *p* = 0.006, Glass’s Δ1 = 0.37, 95% CI [0.12, 0.62]).

There was no significant difference in tumor diameter between spayed dogs (*n* = 168; mean = 3.11; SD = 3.79 cm) and unspayed female dogs (*n* = 498; mean = 3.80; SD = 4.10 cm) (diff. = 0.69 *t*-test = 1.93, *p* = 0.054). 

As for size, 38, 32, and 69 tumors were classified as T1, T2, and T3, respectively [25]. Among the histopathological reports available, only 17.3% (78/449) of the cases provided information on regional lymph nodes. Among these cases, 69.2% (54/78) had compromised regional lymph nodes, while 30% (24/78) showed no evidence of tumor cells.

Staging analysis revealed that 26% (20/78) of female dogs were in stage I (T < 3 cm, N0M0), 2.5% (2/78) in stage II (T: 3–5 cm N0M0), 2.5% (2/78) in stage III (T > 5 cm N0M0), 61% (48/78) in stage IV (N1M0), and 8% (6/78) of the female dogs were stage V (M1) of the disease (Table 2). 

Tumors considered stage I were smaller in diameter (*n* = 20; median 1, ranges 0.3–11 cm) than those in stages IV (*n* = 48; median 3.75, ranges 0.6–23 cm) and V (*n* = 6; median 7.75, ranges 2.7–25 cm) (Kruskal–Wallis χ² = 23.691, *p* = 0.000; post hoc test, *p* = 0.001) (↋2 = 0.308 V) [39,40].

### 3.3. Univariate Analysis

The study enrolled 909 female dogs that satisfied the predefined inclusion criteria. Among these cases, 449 were assigned to the case group (female dogs with malignant mammary tumors), while 460 belonged to the control group (female dogs without any type of tumor). The dichotomous dependent variable in this study was the presence or absence of a malignant mammary tumor (yes, case group; no, control group), a factor examined together with other variables.

The preliminary univariate analysis of the variables revealed that the size of the dog, breed type, housing, and body score were significantly associated with the histopathological outcome of malignant mammary tumors (Table 3 and Table 4).

### 3.4. Descriptive Statistics

#### 3.4.1. Age 

The age of the female dogs was assessed at the time of diagnosis, ranging from 3 to 20 years, with a mean age was 10.35 ± 3.030 (mean ± SD). No statistical differences were observed in age between female dogs with malignant mammary tumors (*n* = 449; mean = 10.35; SD = 3.030) and tumor-free female dogs (*n* = 460; mean = 10.15; SD = 2.90). The age distribution of the female dog was evaluated and showed that most of them were between 9 and 12 years at the time of diagnosis, accounting for 47.3% (196/414) of the cases. Furthermore, Poodles (*n* = 60; mean = 10.88; SD = 3.53) were significantly older at the time of diagnosis than other breeds (*n* = 169; mean = 9.91; SD = 2.90) (Diff. = 109; *t*-test = 2.24, *p* = 0.026).

#### 3.4.2. Breed and Size Dog

The present study revealed that small dogs (265/449; 59%) constituted the majority of the female canine population, followed by medium-sized dogs (130/449; 29%) and large dogs (54/449; 12%). Additionally, the size dog was significantly associated with the diagnosis of a malignant mammary tumor (χ² (2) = 16.272; *p* < 0.001).

The percentage of dogs with a defined breed (229/449; 51%) was slightly higher compared to the percentage of dogs without a defined breed (220/449; 48.9%). Among the defined breed, the Poodle was the most common (60/229; 27.4%). However, the power of effect of this result was considered small (Cohen’s ds = 0.34, 95% CI [−0.13, −1.24]) [37,38]. At the time of diagnosis, the mean age of dogs of a specific breed did not differ from the mean age of dogs of an unspecified breed (*t*-test = 1.12, *p* = 0.263). 

#### 3.4.3. Housing

Regarding housing data, 90.4% (*n* = 406) of the cases with malignant mammary tumors had information about housing in medical records. The vast majority of dogs resided indoors (317/406; 78%), while 21.9% (89/406) reside outdoors or on a farm. The type of housing was significantly associated with the diagnosis of malignant mammary tumor (χ² (1) = 26.390; *p* < 0.001).

#### 3.4.4. Body Score

In their medical records, 75.9% (*n* = 341) of the dogs had a body score ranging from 1 to 9/9. Most of the dogs (145/341; 42.5%) were at the ideal weight, followed by overweight (127/341; 37.2%) and obese female dogs (50/341; 14.6%). The body score category was significantly associated with the diagnosis of a malignant mammary tumor (χ² (3) = 16.083; *p* < 0.001).

#### 3.4.5. Reproductive Status

Among female dogs with malignant mammary tumors, 432 had reproductive status information in their medical records, of which 25.6% (111/432) were recorded as spayed. Upon analysis of the age of castration, most spays occurred during adulthood (over four years), with a mean age of castration of 13.08 ± 1.90 years. Only seven female dogs did not have any information in their medical records that would allow identification of their age of castration but were considered not spayed for matching. Most of the dogs (321/432; 74.3%) were not spayed; of these, 43.6% (140/321) were spayed at the time of mastectomy, thus being categorized as not spayed.

### 3.5. Multivariate Analysis and Creation of the Prediction Model

The multivariate logistic regression analysis considered the following variables as risk factors for the development of malignant mammary tumors: size of dog, breed type, housing, and body score (Table 5). The type of diet was not significant, and its exclusion from the model improved its predictive capacity. The model with dog size, breed type, housing, and body score was significant [χ² (15) = 61.863; *p* < 0.001, R2Negelkerke = 0.106]. The Hosmer-Lemeshow test indicated that the model had a good predictive capacity, with a χ² of 2.418 and eight degrees of freedom (*p* = 0.965).

**Figure 2:**
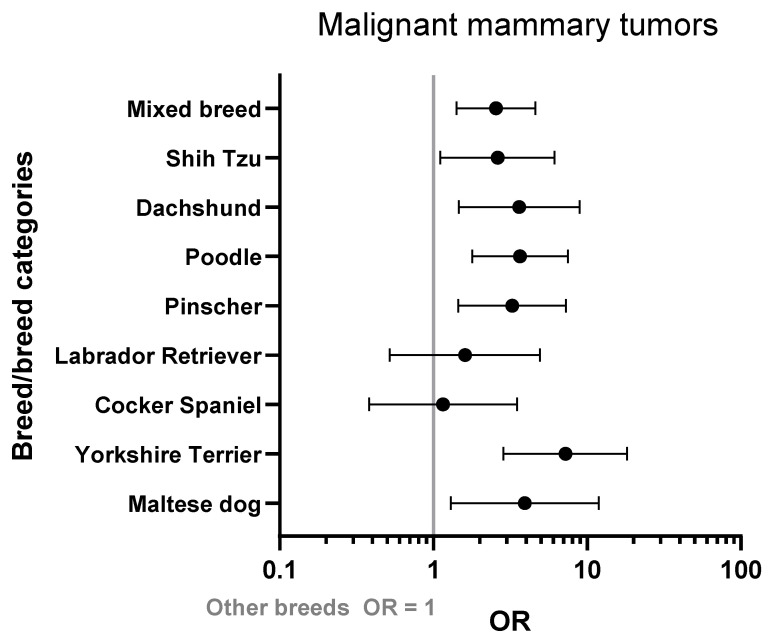
Odds ratios (ORs) and 95% confidence intervals for breeds that commonly developed malignant mammary tumors compared with other breeds (OR = 1).

Size dog, body score, housing, and breed type were confirmed as risk factors. Specifically, the Yorkshire terrier breed was strongly associated with malignant mammary tumors (OR = 7.22, 95% CI: 2.85–18.25, *p* < 0001), but other breeds were also at increased risk, such as Maltese and Poodles (Figure 2). Overweight dogs are at higher risk than animals at the ideal weight (OR = 1.71, 95% CI: 1.20–243, *p* = 0.003).

Size dog was confirmed as a risk factor, with large dogs exhibiting the highest risk (OR = 2.81, 95% CI: 1.46–5.40, *p* = 0.002). Dogs housed outdoors or on the farm are at increased risk of developing malignant mammary tumors (OR = 2.05, 95% CI: 1.30–3.22, *p* = 0.002) compared to those housed indoors. The type of diet was not significantly related to the malignant mammary tumor.

## 4. Discussion

The increasing incidence of neoplasms in dogs is becoming a significant concern in veterinary medicine. Hence, the attention of veterinary oncology has been directed toward the prevention, early diagnosis, and enhancement of the lifespan of dogs with mammary tumors (MTs). In this context, the study of canine cancer is primarily focused on the identification and control of risk factors [8,9,45]. Therefore, a comprehensive retrospective statistical analysis was conducted to determine the risk factors that lead to the development of malignant mammary tumors in dogs. 

The high rate of malignancy found is in line with other studies carried out in Brazil [10,11,12,13,14,15,16,17,18]. One explanation is that early detection and preventive care are still not within the desirable range, and many tumors are diagnosed late. Late diagnosis is related to larger tumors, worse prognosis, and distant metastases [11,13,36,46,47,48]. Furthermore, the time elapsed between the appearance of the lesion and histopathological examination may also contribute to this phenomenon since prolonged time allows the progression of benign to malignant tumors [22]. 

In the present study, many animals were in an advanced stage of the disease; that is, the search for care was late. Socioeconomic factors, such as education and income, made the time to search for veterinary medical services longer [13]. Such observation probably occurred in the studied population since the study’s veterinary hospital is a university hospital and has assistance programs for the low-income population receiving different socioeconomic levels and many animals rescued by nongovernmental organizations (NGOs). These data reinforce the need to be aware of the prevention and early detection of mammary tumors in female dogs at all socioeconomic levels. 

The increase in malignancy is an aspect to be acknowledged and is also observed in human breast cancer [4,9,33]. The rise in malignancy could also be attributed to environmental factors that expose organisms to oncogenic agents. Dogs share the same environment with their guardians and are exposed to the same environmental contaminants, making them potential sentinels [9,49].

The most common type of tumors observed were mixed tumors, which is consistent with the findings reported in the literature. These mixed neoplasms in dogs typically consist of luminal epithelial and interstitial myoepithelial components, often accompanied by areas of mesenchymal tissue, including cartilage, bone, and fat [31,50].

The data presented in the current study support previous findings in the literature on the preferred treatment for mammary tumors in dogs. Except for inflammatory carcinoma, surgical excision (mastectomy or lumpectomy) remains the treatment of choice for MTs [32,51]. In the current study, most dogs underwent surgical removal of their tumors, with an additional 31% of the animals undergoing spaying in conjunction with mastectomy. It is worth noting that spaying female dogs during tumor removal does not impact their survival rates. However, there is a significant decrease in the risk of developing new tumors, which highlights the positive association between castration and mammary tumor removal [52,53].

The study revealed that a large proportion of female dogs with mammary tumors (321 out of 432, representing 74.3%) were not spayed. For those who did undergo spaying, the procedure was performed during adulthood, confirming the protective effects of ovariohysterectomy (OHE) in preventing the development of mammary tumors, as previously described by several authors. These protective effects are likely due to the inhibition of sex steroid effects [23,51,54]. Early neutering, before the first estrous cycle, was routinely recommended for the prevention of mammary cancer in female dogs, as there is substantial evidence linking estrogen with mammary carcinogenesis [23,51,54,55]. As estrogens are also crucial for the proper functioning of numerous organs, scientists are currently questioning this recommendation [56]. Some harmful effects, such as urinary incontinence, skeletal muscle disorders, brain aging, and the development of certain tumors, have been associated with early neutering [54,57,58,59,60,61,62].

It is possible that the beneficial impact of OHE on the prevention of the development of MTs is underestimated and is not supported by consistent scientific data [63,64]. Consequently, there has been an increase in the number of studies that have aimed to determine the optimal age for recommending castration. Identifying animals with a higher risk of developing mammary cancer, particularly the more aggressive types, is crucial. For those at risk, early castration may be beneficial, despite possible long-term side effects [5]. Conversely, it is also essential to identify animals for which the adverse effects of this intervention outweigh the protective effects. 

The vast majority of cases (265/449; 59%) were diagnosed in small dogs. The breeds most commonly diagnosed with mammary tumors were Poodle, Pinscher, Dachshund, Yorkshire, and Shih Tzu. The highest risk was associated with Yorkshire and Poodle breeds. Studies of mammary tumor incidence differ in high-risk breeds depending on the type of study and geographic location, but Poodles and Yorkshires are often among the highest-risk breeds, as demonstrated in this study [4,33,65,66].

The risk of developing mammary tumors in specific breeds may be influenced by hereditary genetic factors. This is because the genetic diversity within a particular breed is generally lower compared to the overall diversity of the entire dog species. Consequently, certain breeds may accumulate risk alleles over time due to limited gene flow. Germline mutations in the BRCA1/2 gene have been linked to the development of mammary tumors in female dogs [66,67,68,69,70]. However, further investigations are necessary to fully understand the role of hereditary genetic components in the development of mammary tumors in female dogs.

In the current study, large dogs had a higher susceptibility to malignant mammary tumors. This association can be attributed to the fact that larger breeds generally have a shorter life expectancy compared to smaller breeds, resulting in accelerated aging. This accelerated aging process leads to increased rates of cell replication, thereby augmenting the risk of replication-associated abnormalities. Furthermore, larger breeds require a greater number of cells due to their larger body size, further contributing to the replicative risk [71,72]. The correlation between increased body size in dogs and an elevated risk of cancer, including mammary tumors, has been documented across various studies [57,73,74,75,76,77]. Despite most of the cases being small size dogs, the greatest risk was associated with large dogs. This finding may be indicative of the canine population distribution in the studied region, which is likely composed mainly of small and medium-sized dogs, as evidenced by the distribution of the control group in our study.

The mean age of the case group was 10.3 years, consistent with other studies for mammary tumors that demonstrate a higher incidence of mammary tumors in dogs between 9–11 years of age [1,8,9,78]. Age demonstrates a strong association with cancer, a fact that can be explained by the chronic selection of cells that stochastically accumulate mutations during normal cell replication, leading to malignant transformation [79]. In fact, longevity is intrinsically associated with cancer risk, but only when this longevity exceeds the evolutionarily adapted lifespan of that species [77,80].

The present study demonstrated that excess weight is an important risk factor for the development of malignant mammary tumors in female dogs. It is hypothesized that the peripheral aromatization of androgens to estrogens occurs at an increased rate in overweight female dogs and can lead to prolonged exposure of mammary tissue to estrogens, especially during puberty. Furthermore, overweight dogs may experience increased insulin levels and higher concentrations of the insulin-like growth factor-1 (IGF-1) protein. These factors have the potential to influence the process of carcinogenesis [25,30,81,82].

In this case-control study, the relationship between eating habits, living environment, and the risk of malignant mammary tumors in female dogs was investigated. The results showed that while diet type did not appear to be a significant risk factor for the development of these tumors, available data on the quantity and quality of the foods consumed may not have been sufficient to fully capture the complexity of the dietary patterns. Conversely, dogs living outside the home had a higher risk of developing a malignant mammary tumor compared to those living indoors, possibly due to reduced access to preventive care and lower levels of attention to their overall health and well-being. These findings highlight the importance of closely monitoring lifestyle factors that can contribute to the occurrence of canine mammary tumors. They also suggested that taking proactive measures to promote healthy living environments and behaviors could serve as an effective approach to reducing this risk.

MTs have a high risk of developing metastases, which occur in about 50% of cases, with lymph node involvement associated with the development of distant metastases in most cases [6,83]. The search for metastasis in regional lymph nodes is a crucial step in tumor staging [22,48]. Since 2010, a Brazilian consensus has been established on the diagnosis, prognosis, and treatment of canine mammary tumors [32]. One of the key recommendations in this consensus is to take the lymphatic system into account during surgical procedures. However, recent research conducted in Brazil revealed that 56.31% of the veterinarians surveyed did not adhere to these consensus recommendations. Additionally, certain laboratories did not follow the specified criteria in the analysis of the samples [47].

This is one of the main problems faced in Brazil since many patients do not have access to complementary tests and do not even benefit from lymph node removal, facts related to the professional lack of knowledge and financial restrictions of owners [47]. In the same trend, in the present study, only 17.1% of females had information about regional lymph nodes in their medical records. During the assessment of medical records, several factors contributed to inadequate planning. The primary contributors were owners’ refusal to undergo further diagnostic tests, financial constraints, and unsatisfactory conduct by the veterinarian or pathologist. These factors can impede proper disease management and lead to suboptimal patient outcomes. The primary objective of establishing an oncology sector in the hospital of this study is to establish an appropriate protocol for all cases of neoplasia in dogs, with the aim of providing effective treatment.

The clinical stage is an important prognostic factor, and female dogs with tumors in advanced stages (IV and V) have a worse prognosis [6,84]. At the time of the study, most of the dogs were in stage IV, in contrast to what has been reported that stages I to III are the most frequent [84,85].

The increase in the number of animals with advanced stages of the disease can be attributed to the lack of a standardized protocol to manage dogs with suspected neoplastic disease prior to 2018. This is corroborated by the fact that only 78 animals had staging information available. Despite the inauguration of the oncology sector in 2018, the implementation of care protocols demands time and staff training. As such, it is probable that only animals with clinically severe tumors had a stage in their medical records, while those with smaller tumors in early stages may not have undergone proper staging or received treatment in the oncology sector.

The diameter of the tumor is a crucial determinant of the prognosis of canines with mammary tumors [85]. Larger tumor diameters of greater than 3.5 cm have a worse prognosis than smaller tumors. This observation is consistent with the results of the present study, in which the average tumor diameter of dogs in stages IV and V was higher than in the other stages. Thus, tumor size serves as a critical parameter to determine the prognosis of mammary tumors, as more aggressive tumors tend to have faster growth rates and larger diameters [6,85].

The significant prevalence of mammary tumors observed in this study highlights the continued significance of this veterinary health concern. It underscores the need for further research to thoroughly analyze the exposure to risk factors associated with mammary tumors in dogs within the specific region under investigation.

A crucial limitation that commonly affects retrospective studies is selection bias, which was unlikely to occur in this study since all cases of malignant mammary tumors were selected during the specified period. However, the paucity of available data was a notable limitation of the present study, as many patients lacked adequate information to facilitate accurate staging.

## 5. Conclusions

The present investigation revealed that certain individual risk factors, including the size dog, breed, housing, and body score, were associated with an elevated susceptibility to the development of malignant mammary tumors in dogs. Specifically, large dog breeds demonstrate a higher proclivity for the disease, while Poodles and Yorkshire Terriers exhibit an increased risk of developing malignant mammary tumors. Additionally, overweight dogs or those who live outdoors were also found to have a greater risk of developing this malignancy. These findings may help in informed decision-making and evaluation of each patient, as well as in the implementation of suitable preventive measures and appropriate therapeutic approaches.

## Figures and Tables

**Table 1:** Frequency of individual subtypes of canine mammary tumors.

Histological Diagnosis		Frequency	%
Simple benign tumors	Adenoma simple	36	4.18
Non-simple benign tumors	Benign mixed tumor	37	4.30
	Fibroadenoma	4	0.46
	Complex adenoma	24	2.79
Ductal-associated benign tumors	Ductal adenoma	2	0.23
	Intraductal papillary adenoma	2	0.23
Carcinoma in situ	Carcinoma in situ	27	3.14
Simple carcinomas	Tubular carcinoma (including *cribriform* carcinoma)	104	12.1
	Tubulopapillary carcinoma	135	15.70
	Solid carcinoma	46	5.34
	Invasive micropapillary carcinoma	15	1.74
	Comedocarcinoma	5	0.58
	Anaplastic carcinoma	9	1.05
Non-simple carcinomas	Mixed carcinoma	207	24.00
	Complex carcinoma	144	16.72
	Carcinoma-and-malignant myoepithelioma	6	0.69
Ductal-associated carcinomas	Intraductal papillary carcinoma (including *papillary-cystic* carcinoma)	5	0.58
Malignant Tumors–Special Types	Squamous cell carcinoma	16	1.86
	Adenosquamous carcinoma	1	0.12
	Mucinous carcinoma	2	0.23
	Lipid-rich carcinoma	2	0.23
	Malignant myoepithelioma *	1	0.12
	Inflammatory mammary carcinoma histological type not specified	4	0.46
Other Tumors Arising at the Site of the Mammary Gland	Osteosarcoma	12	1.40
	Chondrosarcoma	7	0.81
Carcinosarcoma	Carcinosarcoma	8	0.93
Total		861	100

* The diagnosis of malignant myoepithelioma was made without immunohistochemistry.

**Table 2:** The distribution of the 78 carcinoma subtypes according to the degrees of malignancy by the modified TNM classification [6,25].

Subtype	Stage I (T1 < 3 cm)	Stage II (T2: 3–5 cm)	Stage III (T3: >5 cm)	Stage IV (_Any_T N1 M0)	Stage V (_Any_T _Any_N M1)
Tubular carcinoma	27% (3/11)	-	9% (1/11)	55% (6/11)	9% (1/11)
Tubulopapillary carcinoma	45% (5/11)	-	-	55% (6/11)	-
Solid carcinoma	25% (2/8)	-	-	75% (6/8)	-
Invasive micropapillary carcinoma	-	-	-	75% (3/4)	25% (1/4)
Anaplastic carcinoma	50% (1/2)	-	-	50% (1/2)	-
Osteosarcoma					100% (1/1)
Chondrosarcoma					100% (1/1)
Mixed carcinoma	31% (7/23)	4% (1/23)	4% (1/23)	57% (13/23)	4% (1/23)
Complex carcinoma	10% (1/10)	10% (1/10)	-	80% (8/10)	-
Lipid-rich carcinoma					100% (1/1)
Intraductal papillary carcinoma (including papillary-cystic carcinoma)	50% (1/2)	-	-	50% (1/2)	-
Inflammatory mammary carcinoma histological type not specified				100% (1/1)	
Carcinosarcoma				100% (1/1)	
Comedocarcinoma				100% (1/1)	
Carcinoma-and-malignant myoepithelioma	-	-	-	100% (1/1)	-
**Total**	20/78	2/78	2/78	48/78	6/78

Abbreviations: T: primary tumor size; N: regional lymph node status; N1: metastasis; N0: no metastasis. M: distant metastasis; M1: distant metastasis detected; M0: no distant metastasis.

**Table 3:** Descriptive analysis of continuous variables for cases and controls.

Factor	Cases (*n =* 449)	Controls (*n =* 460)	*p*-Value *
Age (years):			0.324
Mean	10.35	10.15	
Std Dev	3.03	2.90	
Weight (Kg):			0.056
Mean	11.74	10.58	
Std Dev	9.26	9.06	

Cases: dogs with malignant mammary tumors; controls: tumor-free dogs. Std Dev: standard deviation; * *p*-Value calculated with *t*-test (age and weight).

**Table 4:** Descriptive analysis of categorical variables for cases and controls.

Factor	Cases (*n =* 449)	Controls (*n =* 460)	*p*-Value *
Age groups			0.593
0–4 years	10	10	
5–8 years	109	125	
9–12 years	196	232	
>13 years	99	93	
Not reported ^+^	35	0	
Size dog:			<0.001
Small	265	330	
Medium	130	91	
Large	54	39	
Body score			<0.001
Underweight	19	39	
Ideal weight	145	220	
Overweight	127	109	
Obesity	50	78	
Not reported ^+^	108	14	
Breed type			0.115
Mixed	220	194	
Poodle	60	69	
Pinscher	32	37	
Dachshund	26	18	
Shihtzu	22	34	
Labrador Retriever	10	10	
Yorkshire terrier	23	15	
Cocker spaniel	10	13	
Maltese dog	10	11	
Other Breeds	36	59	
Hosing			<0.001
Inside home	317	417	
Outside home/farm	89	43	
Not reported ^+^	43	0	
Type of diet:			0.848
Homemade food	17	22	
Dry food	180	193	
Homemade and dry food	228	245	
Not reported ^+^	24	0	

Cases: dogs with malignant mammary tumors; controls: tumor-free dogs. Std Dev: standard deviation; ^+^ Unreported data were not included in the analyses; * *p*-value calculated with chi-square test.

**Table 5:** Multivariable logistic regression model analysis comparing cases and controls group for malignant mammary tumors.

Factor	Odds Ratio (OR)	95 % CIs	*p*-Value
Body score			
Ideal weight (Ref)			
Underweight	0.82	0.44–1.52	0.355
Overweight	1.71	1.20–2.43	0.003
Obesity	0.84	0.53–1.33	0.465
Size dog			
Small (Ref)			
Medium	2.26	1.48–3.44	<0.001
Large	2.81	1.46–5.40	0.002
Breed type			
Other Breeds (Ref)			
Mixed	2.55	1.41–4.61	0.002
Poodle	3.66	1.79–7.51	<0.001
Pinscher	3.25	1.45–7.29	0.004
Dachshund	3.62	1.46–8.94	0.005
Shihtzu	2.62	1.11–6.15	0.027
Labrador Retriever	1.61	0.52–4.93	0.405
Yorkshire terrier	7.22	2.85–18.25	<0.001
Cocker spaniel	1.21	0.39–3.73	0.738
Maltese dog	3.93	1.30–11.87	0.015
Housing			
Inside home (Ref)			
Outside home/farm	2.05	1.30–3.22	0.002

Ref: reference category.

## Data Availability

Not applicable.

## References

[1] Egenvall A., Bonnett B.N., Öhagen P., Olson P., Hedhammar Å., von Euler H. (2005). Incidence of and Survival after Mammary Tumors in a Population of over 80,000 Insured Female Dogs in Sweden from 1995 to 2002. Prev. Vet. Med..

[2] Merlo D.F., Rossi L., Pellegrino C., Ceppi M., Cardellino U., Capurro C., Ratto A., Sambucco P.L., Sestito V., Tanara G. (2008). Cancer Incidence in Pet Dogs: Findings of the Animal Tumor Registry of Genoa, Italy. J. Vet. Intern. Med..

[3] Brønden L.B., Nielsen S.S., Toft N., Kristensen A.T. (2010). Data from the Danish Veterinary Cancer Registry on the Occurrence and Distribution of Neoplasms in Dogs in Denmark. Vet. Rec..

[4] Salas Y., Márquez A., Diaz D., Romero L. (2015). Epidemiological Study of Mammary Tumors in Female Dogs Diagnosed during the Period 2002-2012: A Growing Animal Health Problem. PLoS ONE.

[5] Canadas A., França M., Pereira C., Vilaça R., Vilhena H., Tinoco F., Silva M.J., Ribeiro J., Medeiros R., Oliveira P. (2019). Canine Mammary Tumors: Comparison of Classification and Grading Methods in a Survival Study. Vet. Pathol..

[6] Sorenmo K.U., Worley D.R., Zappulli V. (2019). Tumors of the Mammary Gland. Withrow and MacEwen’s Small Animal Clinical Oncology.

[7] Dorn C.R. (1967). The Epidemiology of Cancer in Animals. Calif. Med..

[8] Grüntzig K., Graf R., Hässig M., Welle M., Meier D., Lott G., Erni D., Schenker N.S., Guscetti F., Boo G. (2015). The Swiss Canine Cancer Registry: A Retrospective Study on the Occurrence of Tumours in Dogs in Switzerland from 1955 to 2008. J. Comp. Pathol..

[9] Vascellari M., Capello K., Carminato A., Zanardello C., Baioni E., Mutinelli F. (2016). Incidence of Mammary Tumors in the Canine Population Living in the Veneto Region (Northeastern Italy): Risk Factors and Similarities to Human Breast Cancer. Prev. Vet. Med..

[10] da Silva A.L., Albinati A.C.L., de Marques J.V.S., de Souza Y.R.C., Maia I.P.C., dos Santos C.L., da Brito V.E.S., da Braga E.S. (2021). Prevalência de neoplasias mamárias em cadelas e gatas no hospital veterinário da Univasf em Petrolina / Mammary neoplasia prevalence in bitches and female cats in the veterinary hospital of Univasf in Petrolina. Braz. J. Vet. Res. Anim. Sci..

[11] Biondi L.R., Gentile L.B., da Rego A.A.M.S., Noronha N.P., Dagli M.L.Z. (2014). Canine Mammary Tumors in Santos, Brazil: Clinicopathological and Survival Profile. Braz. J. Vet. Res. Anim. Sci..

[12] Oliveira Filho J.C., Kommers G.D., Masuda E.K., Marques B.M.F.P.P., Fighera R.A., Irigoyen L.F., Barros C.S.L. (2010). Estudo retrospectivo de 1.647 tumores mamários em cães. Pesq. Vet. Bras..

[13] de Toríbio J.M.M.L., Lima A.E., Martins Filho E.F., Ribeiro L.G.R., D’Assis M.J.M.H., Teixeira R.G., Damasceno K.A., Cassali G.D., da Costa Neto J.M. (2012). Caracterização Clínica, Diagnóstico Histopatológico e Distribuição Geográfica Das Neoplasias Mamárias Em Cadelas de Salvador, Bahia. Rev. Ceres.

[14] De Nardi A.B., Rodaski S., Sousa R.S., Costa T.A., Macedo T.R., Rodigheri S.M., Rios A., Piekarz C.H. (2002). Prevalência de neoplasias e modalidades de tratamentos em cães, atendidos no Hospital Veterinário da Universidade Federal do Paraná. Arch. Vet. Sci..

[15] Bentubo H.D.L., Tomaz M.A., Bondan E.F., Lallo M.A. (2007). Expectativa de vida e causas de morte em cães na área metropolitana de São Paulo (Brasil). Cienc. Rural.

[16] de Oliveira L.O., de Oliveira R.T., Loretti A.P., Rodrigues R., Driemeier D. (2018). Aspectos Epidemiológicos Da Neoplasia Mamária Canina. Acta Sci. Vet..

[17] Da Martins A.M.C.R.P., Tamaso E., Guerra J.L. (2002). Retrospective Review and Systematic Study of Mammary Tumors in Dogs and Characteristics of the Extracellular Matrix. Braz. J. Vet. Res. Anim. Sci..

[18] Oliveira L.C., dos Fernandes M.E.S.L., Peixoto A.J.R., da Barros F.F.P.C., Coelho C.M.M., de Nogueira V.A., Caldas S.A. (2022). Clinical, Epidemiological, and Histopathological Aspects of Breast Cancer in Female Dogs at Federal Rural University of Rio de Janeiro Veterinary Hospital. Braz. J. Vet. Med..

[19] MacEwen E.G. (1990). Spontaneous Tumors in Dogs and Cats: Models for the Study of Cancer Biology and Treatment. Cancer Metastasis Rev..

[20] Vail D.M., Macewen E.G. (2000). Spontaneously Occurring Tumors of Companion Animals as Models for Human Cancer. Cancer Investig..

[21] Raditic D.M., Bartges J.W. (2014). Evidence-Based Integrative Medicine in Clinical Veterinary Oncology. Vet. Clin. N. Am. Small Anim. Pract..

[22] Sorenmo K.U., Kristiansen V.M., Cofone M.A., Shofer F.S., Breen A.-M., Langeland M., Mongil C.M., Grondahl A.M., Teige J., Goldschmidt M.H. (2009). Canine Mammary Gland Tumours; a Histological Continuum from Benign to Malignant; Clinical and Histopathological Evidence. Vet. Comp. Oncol..

[23] Schneider R., Dorn C.R., Taylor D.O. (1969). Factors Influencing Canine Mammary Cancer Development and Postsurgical Survival. J. Natl. Cancer. Inst..

[24] Taylor G.N., Shabestari L., Williams J., Mays C.W., Angus W., McFarland S. (1976). Mammary Neoplasia in a Closed Beagle Colony. Cancer Res..

[25] Sorenmo K.U., Rasotto R., Zappulli V., Goldschmidt M.H. (2011). Development, Anatomy, Histology, Lymphatic Drainage, Clinical Features, and Cell Differentiation Markers of Canine Mammary Gland Neoplasms. Vet. Pathol..

[26] Grüntzig K., Graf R., Boo G., Guscetti F., Hässig M., Axhausen K.W., Fabrikant S., Welle M., Meier D., Folkers G. (2016). Swiss Canine Cancer Registry 1955–2008: Occurrence of the Most Common Tumour Diagnoses and Influence of Age, Breed, Body Size, Sex and Neutering Status on Tumour Development. J. Comp. Pathol..

[27] Carmichael A.R., Bates T. (2004). Obesity and Breast Cancer: A Review of the Literature. Breast.

[28] Lee K., Kruper L., Dieli-Conwright C.M., Mortimer J.E. (2019). The Impact of Obesity on Breast Cancer Diagnosis and Treatment. Curr. Oncol. Rep..

[29] Sonnenschein E.G., Glickman L.T., Goldschmidt M.H., McKee L.J. (1991). Body Conformation, Diet, and Risk of Breast Cancer in Pet Dogs: A Case-Control Study. Am. J. Epidemiol..

[30] Lim H.-Y., Seung B.-J., Cho S.-H., Kim S.-H., Bae M.-K., Sur J.-H. (2022). Canine Mammary Cancer in Overweight or Obese Female Dogs Is Associated with Intratumoral Microvessel Density and Macrophage Counts. Vet. Pathol..

[31] Zappulli V., Pena L., Rassotto R., Goldschmidt M.H., Gama A., Scruggs J.L., Kiupel M., Kiupel M. (2019). Mammary Tumors, Surgical Pathology of Tumors of Domestic Animals.

[32] Cassali G.D., Jark P., Gamba C., Damasceno K., Estrela-Lima A., Nardi A., Ferreira E., Horta R., Firmo B., Sueiro F. (2020). Consensus Regarding the Diagnosis, Prognosis and Treatment of Canine and Feline Mammary Tumors—2019. Braz. J. Vet. Pathol..

[33] Rasotto R., Zappulli V., Castagnaro M., Goldschmidt M.H. (2012). A Retrospective Study of Those Histopathologic Parameters Predictive of Invasion of the Lymphatic System by Canine Mammary Carcinomas. Vet. Pathol..

[34] Peña L., Andrés P.J.D., Clemente M., Cuesta P., Pérez-Alenza M.D. (2013). Prognostic Value of Histological Grading in Noninflammatory Canine Mammary Carcinomas in a Prospective Study with Two-Year Follow-Up: Relationship With Clinical and Histological Characteristics. Vet. Pathol..

[35] Owen L.N. (1980). TNM Classification of Tumours in Domestic Animals.

[36] Santos T.R. (2018). dos Implantação Do Serviço de Oncologia Veterinária No Hospital Veterinário Da Universidade Federal de Uberlândia. Doutorado em Ciências Veterinárias, Universidade Federal de Uberlândia, Uberlândia. https://repositorio.ufu.br/handle/123456789/23156.

[37] Cohen J. (2009). Statistical Power Analysis for the Behavioral Sciences.

[38] Sawilowsky S.S. (2009). New Effect Size Rules of Thumb. J. Mod. App. Stat. Meth..

[39] King B.M., Rosopa P.J., Minium E.W. (2018). Statistical Reasoning in the Behavioral Sciences.

[40] Mangiafico S.S. Summary and Analysis of Extension Program Evaluation in R. https://rcompanion.org/documents/RHandbookProgramEvaluation.pdf.

[41] Laflamme D. (1997). Development and validation of a body condition score system for dogs. Canine Pract..

[42] Grandjean D. (2006). The Dog Encyclopedia.

[43] Hosmer D.W., Lemeshow S. (1989). Applied Logistic Regression.

[44] Hosmer D.W., Lemeshow S., Sturdivant R.X. (2013). Applied Logistic Regression.

[45] Ruple A., Bonnett B.N., Page R.L., Vail D.M., Thamm D.H., Liptak J.M. (2020). Epidemiology and the Evidence-Based Medicine Approach. Withrow and MacEwen’s Small Animal Clinical Oncology.

[46] de Dias M.L.M., Andrade J.M.L., de Castro M.B., Galera P.D. (2016). Survival Analysis of Female Dogs with Mammary Tumors after Mastectomy: Epidemiological, Clinical and Morphological Aspects. Pesq. Vet. Bras..

[47] Zuchi T., Lopatini C., Faria J. (2021). Veterinary Approaches to Canine Mammary Tumors and Knowledge of the Consensus Statement in Brazil. Braz. J. Vet. Pathol..

[48] Sorenmo K. (2003). Canine Mammary Gland Tumors. Vet. Clin. N. Am. Small Anim. Pract..

[49] Jenkins S., Betancourt A.M., Wang J., Lamartiniere C.A. (2012). Endocrine-Active Chemicals in Mammary Cancer Causation and Prevention. J. Steroid Biochem. Mol. Biol..

[50] Cassali G.D., Cavalheiro Bertagnolli A., Ferreira E., Araújo Damasceno K., de Oliveira Gamba C., Bonolo de Campos C. (2012). Canine Mammary Mixed Tumours: A Review. Vet. Med. Int..

[51] dos Horta R.S., Lavalle G.E., de Cunha R.M.C., de Moura L.L., de Araújo R.B., Cassali G.D. (2014). Influence of Surgical Technique on Overall Survival, Disease Free Interval and New Lesion Development Interval in Dogs with Mammary Tumors. Adv. Breast Cancer Res..

[52] Kristiansen V.M., Nødtvedt A., Breen A.M., Langeland M., Teige J., Goldschmidt M., Jonasdottir T.J., Grotmol T., Sørenmo K. (2013). Effect of Ovariohysterectomy at the Time of Tumor Removal in Dogs with Benign Mammary Tumors and Hyperplastic Lesions: A Randomized Controlled Clinical Trial. J. Vet. Intern. Med..

[53] Kristiansen V.M., Peña L., Díez Córdova L., Illera J.C., Skjerve E., Breen A.M., Cofone M.A., Langeland M., Teige J., Goldschmidt M. (2016). Effect of Ovariohysterectomy at the Time of Tumor Removal in Dogs with Mammary Carcinomas: A Randomized Controlled Trial. J. Vet. Intern. Med..

[54] Zink M.C., Farhoody P., Elser S.E., Ruffini L.D., Gibbons T.A., Rieger R.H. (2014). Evaluation of the Risk and Age of Onset of Cancer and Behavioral Disorders in Gonadectomized Vizslas. J. Am. Vet. Med. Assoc..

[55] Burrai G.P., Gabrieli A., Moccia V., Zappulli V., Porcellato I., Brachelente C., Pirino S., Polinas M., Antuofermo E. (2020). A Statistical Analysis of Risk Factors and Biological Behavior in Canine Mammary Tumors: A Multicenter Study. Animals.

[56] Sundburg C.R., Belanger J.M., Bannasch D.L., Famula T.R., Oberbauer A.M. (2016). Gonadectomy Effects on the Risk of Immune Disorders in the Dog: A Retrospective Study. BMC Vet. Res..

[57] Ru G., Terracini B., Glickman L.T. (1998). Host Related Risk Factors for Canine Osteosarcoma. Vet. J..

[58] Cooley D.M., Beranek B.C., Schlittler D.L., Glickman N.W., Glickman L.T., Waters D.J. (2002). Endogenous Gonadal Hormone Exposure and Bone Sarcoma Risk. Cancer Epidemiol. Biomark. Prev..

[59] de la Torres Riva G., Hart B.L., Farver T.B., Oberbauer A.M., McMessam L.L.V., Willits N., Hart L.A. (2013). Neutering Dogs: Effects on Joint Disorders and Cancers in Golden Retrievers. PLoS ONE.

[60] Smith A.N. (2014). The Role of Neutering in Cancer Development. Vet. Clin. N. Am. Small Anim. Pract..

[61] Rzechorzek N.M., Saunders O.M., Hiscox L.V., Schwarz T., Marioni-Henry K., Argyle D.J., Schoenebeck J.J., Freeman T.C. (2019). Network Analysis of Canine Brain Morphometry Links Tumour Risk to Oestrogen Deficiency and Accelerated Brain Ageing. Sci. Rep..

[62] Hart B.L., Hart L.A., Thigpen A.P., Willits N.H. (2020). Assisting Decision-Making on Age of Neutering for 35 Breeds of Dogs: Associated Joint Disorders, Cancers, and Urinary Incontinence. Front. Vet. Sci..

[63] Beauvais W., Cardwell J.M., Brodbelt D.C. (2012). The Effect of Neutering on the Risk of Mammary Tumours in Dogs—A Systematic Review. J. Small Anim. Pract..

[64] Arlt S., Wehrend A., Reichler I.M. (2017). Kastration der Hündin—Neue und alte Erkenntnisse zu Vor- und Nachteilen. Tierarztl. Prax. Ausg. K.

[65] Nam A.-R., Lee K.-H., Hwang H.-J., Schabort J.J., An J.-H., Won S.-H., Cho J.-Y. (2020). Alternative Methylation of Intron Motifs Is Associated with Cancer-Related Gene Expression in Both Canine Mammary Tumor and Human Breast Cancer. Clin. Epigenet..

[66] Rivera P., Melin M., Biagi T., Fall T., Häggström J., Lindblad-Toh K., von Euler H. (2009). Mammary Tumor Development in Dogs Is Associated with BRCA1 and BRCA2. Cancer Res..

[67] Klopfleisch R., Gruber A.D. (2009). Increased Expression of BRCA2 and RAD51 in Lymph Node Metastases of Canine Mammary Adenocarcinomas. Vet. Pathol..

[68] Yordy J., Kraus C., Hayward J.J., White M.E., Shannon L.M., Creevy K.E., Promislow D.E.L., Boyko A.R. (2020). Body Size, Inbreeding, and Lifespan in Domestic Dogs. Conserv. Genet..

[69] Yoshikawa Y., Morimatsu M., Ochiai K., Ishiguro-Oonuma T., Wada S., Orino K., Watanabe K. (2015). Reduced Canine BRCA2 Expression Levels in Mammary Gland Tumors. BMC Vet. Res..

[70] Thumser-Henner P., Nytko K.J., Rohrer Bley C. (2020). Mutations of BRCA2 in Canine Mammary Tumors and Their Targeting Potential in Clinical Therapy. BMC Vet. Res..

[71] Sarver A.L., Makielski K.M., DePauw T.A., Schulte A.J., Modiano J.F. (2022). Increased Risk of Cancer in Dogs and Humans: A Consequence of Recent Extension of Lifespan beyond Evolutionarily Determined Limitations?. Aging Cancer.

[72] Ostrander E.A., Dreger D.L., Evans J.M. (2019). Canine Cancer Genomics: Lessons for Canine and Human Health. Annu. Rev. Anim. Biosci..

[73] Pastor N., Caballé N.C., Santella M., Ezquerra L.J., Tarazona R., Duran E. (2018). Epidemiological Study of Canine Mammary Tumors: Age, Breed, Size and Malignancy. Austral. J. Vet. Sci..

[74] Galis F., Van Der Sluijs I., Van Dooren T.J.M., Metz J.A.J., Nussbaumer M. (2007). Do Large Dogs Die Young?. J. Exp. Zool..

[75] Fleming J.M., Creevy K.E., Promislow D.E.L. (2011). Mortality in North American Dogs from 1984 to 2004: An Investigation into Age, Size-, and Breed-Related Causes of Death: Mortality of Dogs in North America. J. Vet. Intern. Med..

[76] Bonnett B.N., Egenvall A., Hedhammar Å., Olson P. (2005). Mortality in over 350,000 Insured Swedish Dogs from 1995-2000: I. Breed, Gender-, Age- and Cause-Specific Rates. Acta Vet. Scand..

[77] Rafalko J.M., Kruglyak K.M., McCleary-Wheeler A.L., Goyal V., Phelps-Dunn A., Wong L.K., Warren C.D., Brandstetter G., Rosentel M.C., DiMarzio L. (2023). Age at Cancer Diagnosis by Breed, Weight, Sex, and Cancer Type in a Cohort of More than 3,000 Dogs: Determining the Optimal Age to Initiate Cancer Screening in Canine Patients. PLoS ONE.

[78] Burrai G.P., Tanca A., De Miglio M.R., Abbondio M., Pisanu S., Polinas M., Pirino S., Mohammed S.I., Uzzau S., Addis M.F. (2015). Investigation of HER2 Expression in Canine Mammary Tumors by Antibody-Based, Transcriptomic and Mass Spectrometry Analysis: Is the Dog a Suitable Animal Model for Human Breast Cancer?. Tumor Biol..

[79] Argyle D.J., Khanna C., Giancristofaro N., Vail D.M., Thamm D.H., Liptak J.M. (2020). Tumor Biology and Metastasis. Withrow and MacEwen’s Small Animal Clinical Oncology.

[80] Nunney L. (2020). Resolving Peto’s Paradox: Modeling the Potential Effects of Size-related Metabolic Changes, and of the Evolution of Immune Policing and Cancer Suppression. Evol. Appl..

[81] Miki Y., Suzuki T., Tazawa C., Yamaguchi Y., Kitada K., Honma S., Moriya T., Hirakawa H., Evans D.B., Hayashi S. (2007). Aromatase Localization in Human Breast Cancer Tissues: Possible Interactions between Intratumoral Stromal and Parenchymal Cells. Cancer Res..

[82] Lim H.Y., Im K.S., Kim N.H., Kim H.W., Shin J.I., Sur J.H. (2015). Obesity, Expression of Adipocytokines, and Macrophage Infiltration in Canine Mammary Tumors. Vet. J..

[83] Gtreee M., Meehan J., Martínez-Pérez C., Kay C., Turnbull A.K., Morrison L.R., Pang L.Y., Argyle D. (2020). Naturally-Occurring Canine Mammary Tumors as a Translational Model for Human Breast Cancer. Front. Oncol..

[84] Nunes F.C., Campos C.B., Teixeira S.V., Bertagnolli A.C., Lavalle G.E., Cassali G.D. (2018). Epidemiological, Clinical and Pathological Evaluation of Overall Survival in Canines with Mammary Neoplasms. Arq. Bras. Med. Vet. Zootec..

[85] MacEwen E.G., Harvey H.J., Patnaik A.K., Mooney S., Hayes A., Kurzman I., Hardy W.D. (1985). Evaluation of Effects of Levamisole and Surgery on Canine Mammary Cancer. J. Biol. Response Mod..

